# Supplementary material for: Recycled arc mantle recovered from the Mid-Atlantic Ridge
Source: Nat Commun. 2020 Aug 4;11:3887. doi: 10.1038/s41467-020-17604-8 (PMC7403410; doi:10.1038/s41467-020-17604-8)
Supplement: Supplementary file 1 — Supplementary Information [file 41467_2020_17604_MOESM1_ESM.pdf]

Supplementary Information

Journal: *Nature Communications*
Title: Recycled arc mantle recovered from the Mid-Atlantic ridge
Authors: Urann et al. 2020

***email: burann@whoi.edu**

Supplementary Figure 1.

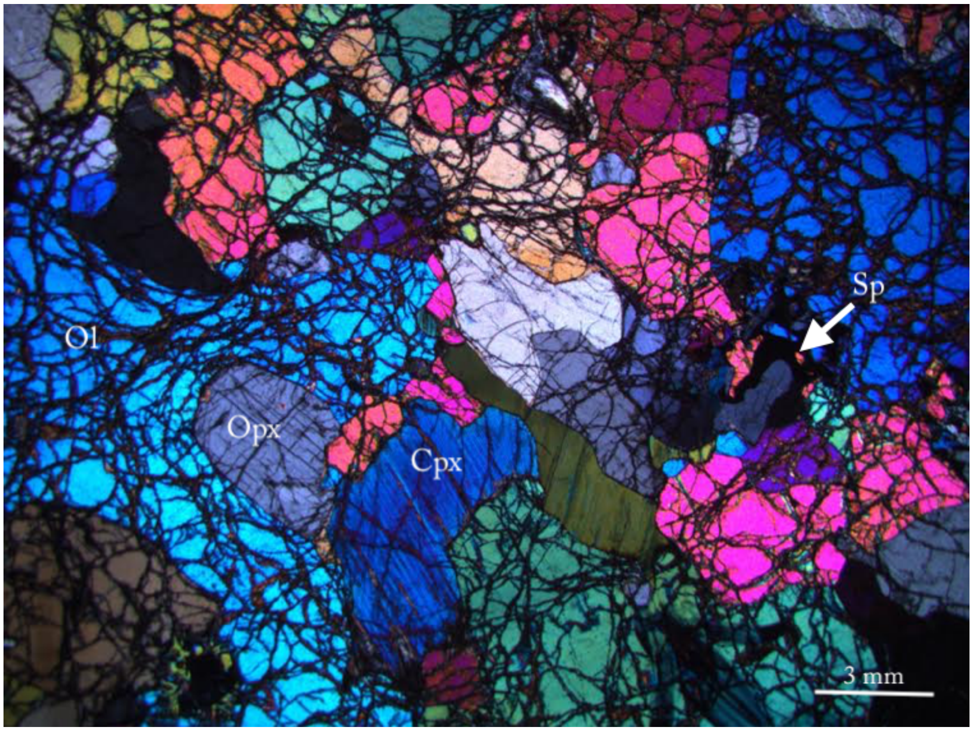

Title: Photomicrograph of exceptionally fresh 16ºN peridotite

Protogranular harzburgite under crossed nicols. Textures are consistent with primary Cpx, not exsolved from Opx. Field of view is 2.5 cm. Ol, Sp, Cpx, Opx are olivine, spinel, clinopyroxene, and orthopyroxene, respectively.

Supplementary Figure 2.

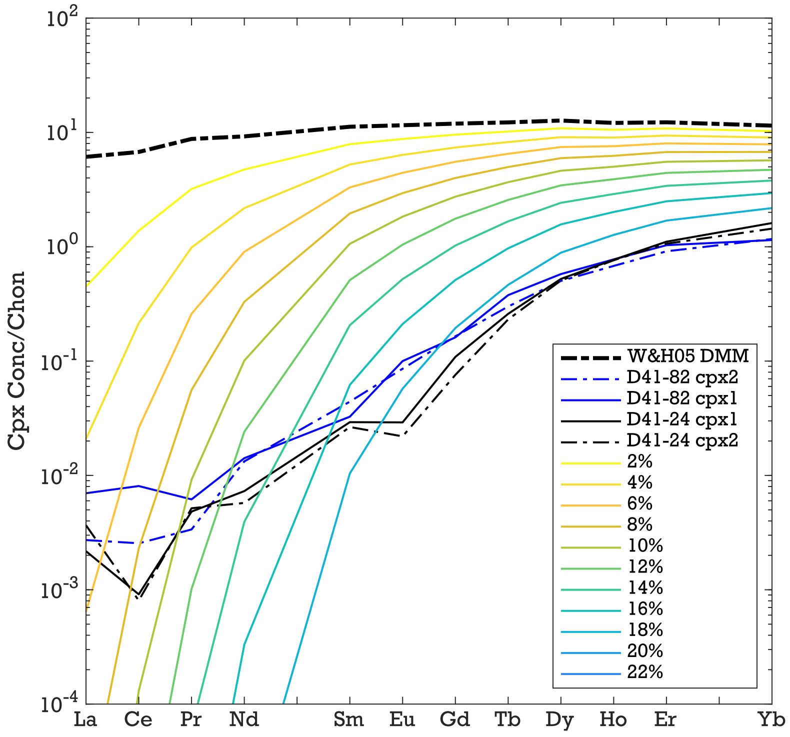

Title: **Fractional melting model of rare earth elements in 16ºN peridotite clinopyroxene**

Spider diagram of REE with anhydrous fractional melting model of spinel-bearing peridotite utilizing MatLab script of Warren[1] and references therein, modified using anhydrous melting modes of Wasylenki et al.[2]. Partition coefficients from Sun and Liang[3]. Note that anhydrous melting is unable to replicate observed REE abundances before Cpx exhaustion. Shown are the median values of five measurements per grain for the samples in this study, normalized to chondrite values of Anders and Grevesse[4]. Thick black dashed line indicates depleted MORB mantle (DMM) values suggested by Workman and Hart[5].

Supplementary Figure 3.

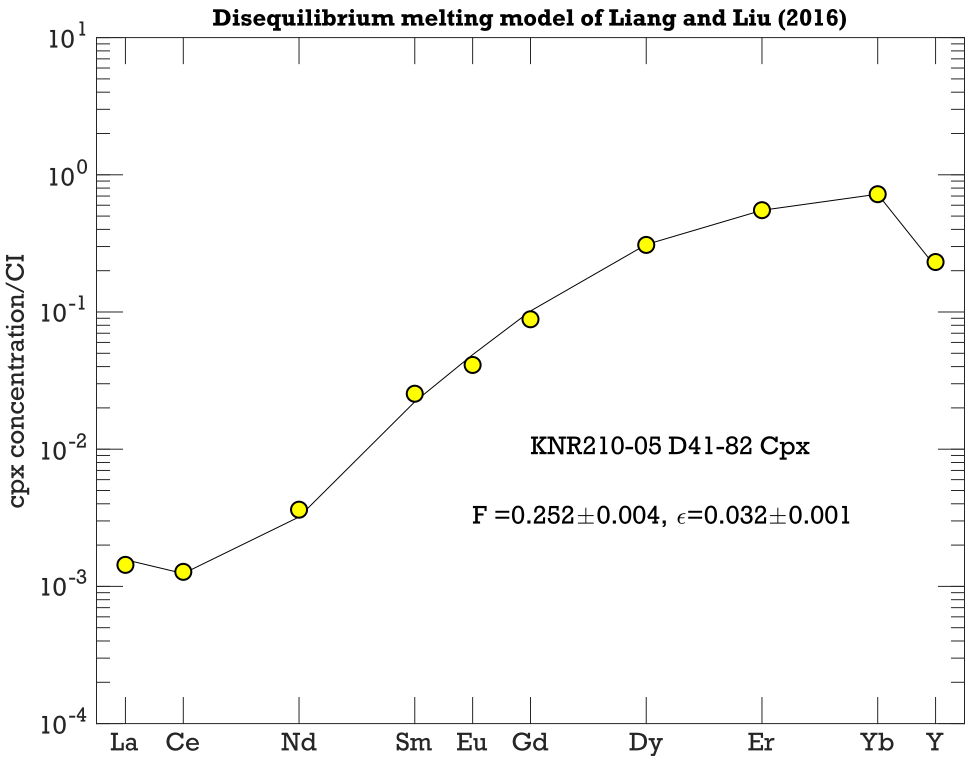

Title: **Disequilibrium melting model of 16ºN clinopyroxene rare earth elements**

REE + Y fractional disequilibrium melting model of Liang and Liu[6], modified to incorporate hydrous melting parameters with starting mineral modal abundances of Workman and Hart[5] DMM. Partition coefficients are from Sun and Liang[3], with hydrous melting modes of Bizimis et al.[7]. Values are normalized to chondrite values of Anders and Grevesse[4].

Supplementary Figure 4.

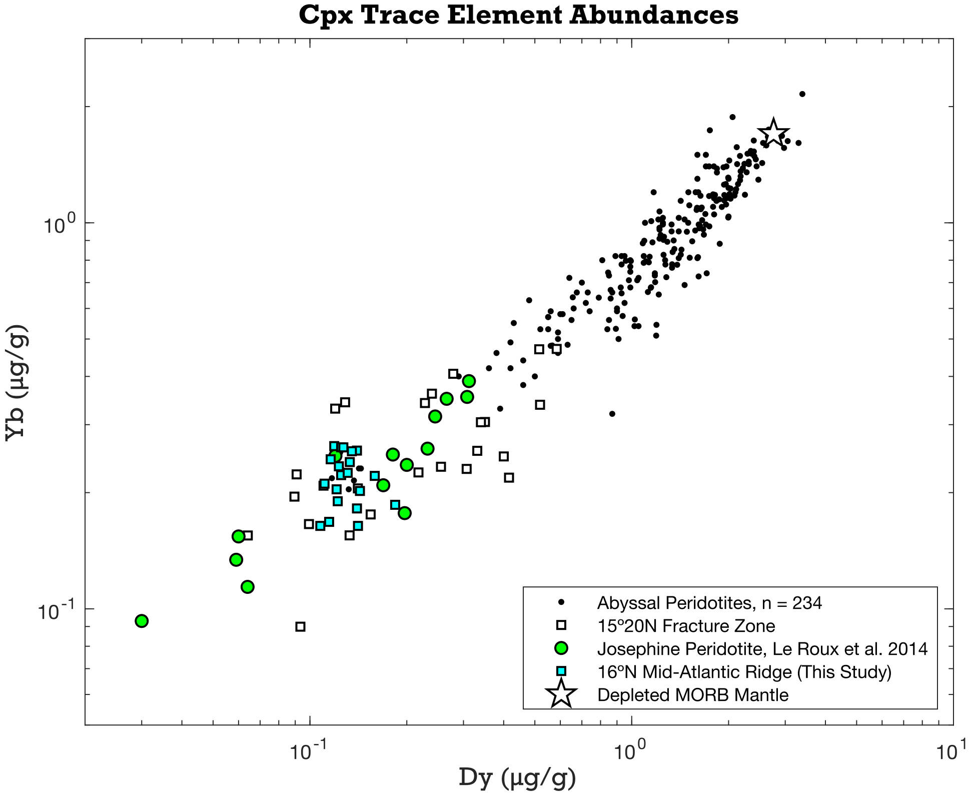

Title: **Heavy rare earth element concentrations from peridotite clinopyroxene**

Clinopyroxene Yb vs. Dy abundances in log-log space, illustrating the highly depleted nature of mantle both in the 16ºN and 15º20'N Fracture Zone regions compared to the abyssal peridotite compilation of Warren[1]. Also plotted is data from an SSZ environment, the Josephine peridotite, for comparison. Josephine peridotite data from Le Roux et al.[8].

Supplementary Figure 5.

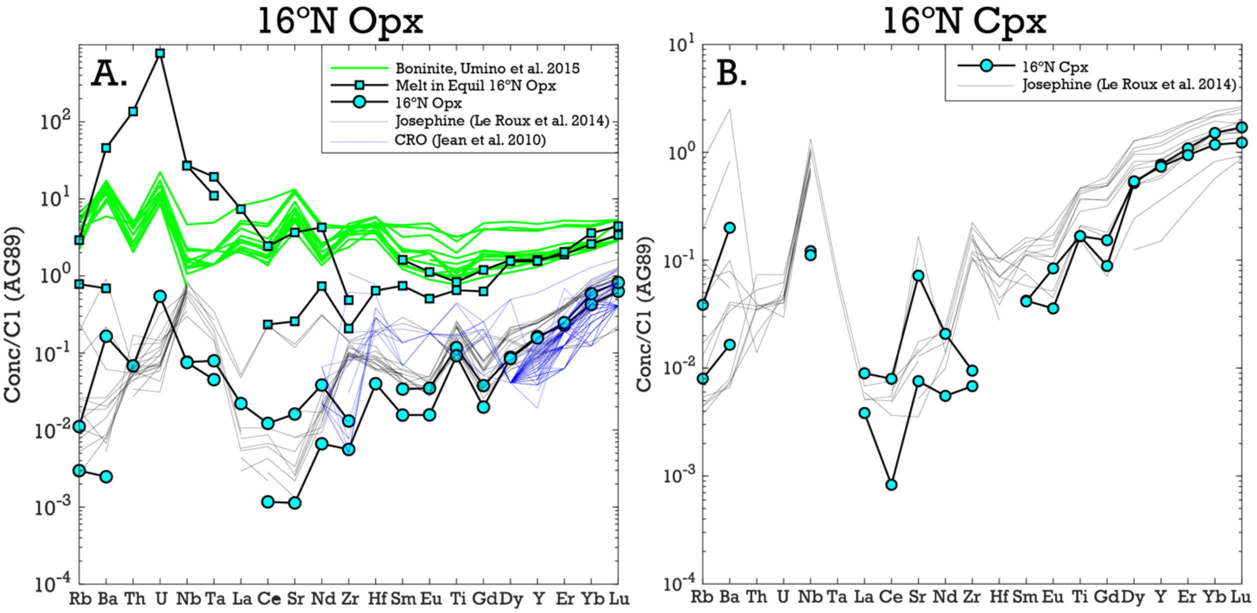

Title: Spider diagram of pyroxene trace element abundances and calculated equilibrium melts

A. Calculated melts in equilibrium with 16ºN Opx (blue squares) as well as 16ºN Opx trace element concentrations (blue circles) normalized to chondritic values of Anders and Grevesse [4]. Calculated melts show extreme fluid immobile element depletion and fluid mobile element enrichments, consistent with flux melting and interaction with boninitic-like melts. B. 16ºN Cpx. Thin lines are literature data from the Josephine peridotite and Coast Range Ophiolite (CRO) peridotites[8,9]. Boninite data from Unimo et al.[10]. Opx-melt partition coefficients from the literature[11,12].

Supplementary Figure 6.

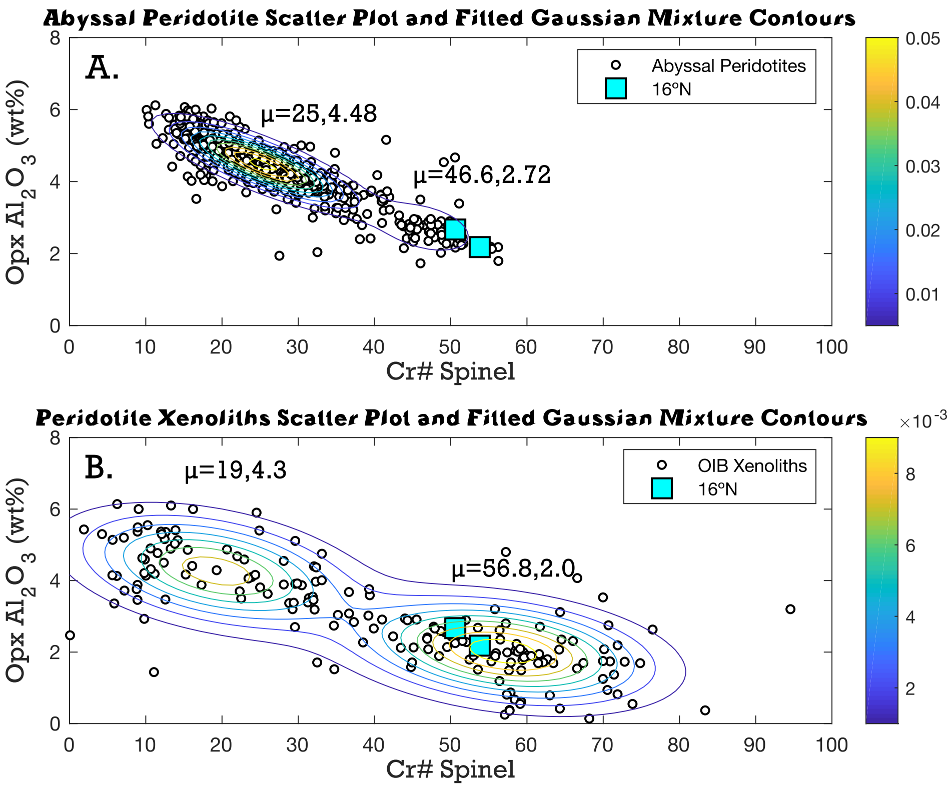

Title: Statistical analysis of abyssal peridotite and ocean island basalt-derived peridotite xenoliths

Probability density contours of Opx Al_2O_3 content and spinel Cr# from A. mid-ocean ridge peridotites (MORP) from Warren et al.[1] and B. ocean island basalt hosted peridotite xenoliths from Simon et al. created using MATLAB Statistics toolbox. Probability contours are color-coded, while mixing calculations suggest two populations with mean values (μ) listed in each figure. We note that abyssal peridotite data show a heavy sampling bias toward fracture zones (~75% of samples) compared to the stochastic sampling of ocean island basalt xenoliths.

Supplementary References

1 Warren, J. M. Global variations in abyssal peridotite compositions. *Lithos* **248**, 193-219, doi:10.1016/j.lithos.2015.12.023 (2016).

2 Wasylenki, L. E., Baker, M. B., Kent, A. J. R. & Stolper, E. M. Near-solidus melting of the shallow upper mantle: Partial melting experiments on depleted peridotite. *J Petrol* **44**, 1163-1191, doi:DOI 10.1093/petrology/44.7.1163 (2003).

3 Sun, C. G. & Liang, Y. Distribution of REE between clinopyroxene and basaltic melt along a mantle adiabat: effects of major element composition, water, and temperature. *Contrib Mineral Petr* **163**, 807-823, doi:10.1007/s00410-011-0700-x (2012).

4 Anders, E. & Grevesse, N. Abundances of the elements: Meteoritic and solar. *Geochim Cosmochim Ac* **53**, 197-214 (1989).

5 Workman, R. K. & Hart, S. R. Major and trace element composition of the depleted MORB mantle (DMM). *Earth Planet Sc Lett* **231**, 53-72, doi:10.1016/j.epsl.2004.12.005 (2005).

6 Liang, Y. & Liu, B. D. Simple models for disequilibrium fractional melting and batch melting with application to REE fractionation in abyssal peridotites. *Geochim Cosmochim Ac* **173**, 181-197, doi:10.1016/j.gca.2015.10.020 (2016).

7 Bizimis, M., Salters, V. J. M. & Bonatti, E. Trace and REE content of clinopyroxenes from supra-subduction zone peridotites. Implications for melting and enrichment processes in island arcs. *Chem Geol* **165**, 67-85, doi:Doi 10.1016/S0009-2541(99)00164-3 (2000).

8 Le Roux, V., Dick, H. J. B. & Shimizu, N. Tracking flux melting and melt percolation in supra-subduction peridotites (Josephine ophiolite, USA). *Contrib Mineral Petr* **168**, doi:ARTN 1064 10.1007/s00410-014-1064-9 (2014).

9 Jean, M. M., Shervais, J. W., Choi, S. H. & Mukasa, S. B. Melt extraction and melt refertilization in mantle peridotite of the Coast Range ophiolite: an LA-ICP-MS study. *Contrib Mineral Petr* **159**, 113-136, doi:10.1007/s00410-009-0419-0 (2010).

10 Umino, S. *et al.* Thermal and chemical evolution of the subarc mantle revealed by spinel-hosted melt inclusions in boninite from the Ogasawara (Bonin) Archipelago, Japan. *Geology* **43**, 151-154, doi:10.1130/G36191.1 (2015).

11 Adam, J. & Green, T. Trace element partitioning between mica-and amphibole-bearing garnet lherzolite and hydrous basanitic melt: 1. Experimental results and the investigation of controls on partitioning behaviour. *Contrib Mineral Petr* **152**, 1-17 (2006).

12 McDade, P., Blundy, J. D. & Wood, B. J. Trace element partitioning on the Tinaquillo Lherzolite solidus at 1.5 GPa. *Physics of the Earth and Planetary Interiors* **139**, 129-147 (2003).
