# Supplementary material for: Recycled arc mantle recovered from the Mid-Atlantic Ridge
Source: Nat Commun. 2020 Aug 4;11:3887. doi: 10.1038/s41467-020-17604-8 (PMC7403410; doi:10.1038/s41467-020-17604-8)
Supplement: Supplementary file 3 — Description of Additional Supplementary Files [file 41467_2020_17604_MOESM3_ESM.pdf]

**Description of Additional Supplementary Files**

File Name: Supplementary Data 1
Description: Major element analyses of olivine, spinel, orthopyroxene, and clinopyroxene

File Name: Supplementary Data 2
Description: Pyroxene trace element concentrations

File Name: Supplementary Data 3
Description: Reference material measurements throughout LA-ICP-MS session

File Name: Supplementary Data 4
Description: Additional peridotite major element analyses, clinopyroxene trace element data

File Name: Supplementary Data 5
Description: Melting model parameters used in this study with references
